# Variation in thermal stress response in two populations of the brown seaweed, *Fucus distichus*, from the Arctic and subarctic intertidal

**DOI:** 10.1098/rsos.150429

**Published:** 2016-01-13

**Authors:** Irina Smolina, Spyros Kollias, Alexander Jueterbock, James A. Coyer, Galice Hoarau

**Affiliations:** 1 Faculty of Biosciences and Aquaculture, University of Nordland, Bodø 8049, Norway; 2 Shoals Marine Laboratory, Cornell University, 400 Little Harbor Road, Portsmouth, NH 03801, USA

**Keywords:** thermal stress response, brown algae, local adaptation, heat shock protein genes, photosynthetic performance

## Abstract

It is unclear whether intertidal organisms are ‘preadapted’ to cope with the increase of temperature and temperature variability or if they are currently at their thermal tolerance limits. To address the dichotomy, we focused on an important ecosystem engineer of the Arctic intertidal rocky shores, the seaweed *Fucus distichus* and investigated thermal stress responses of two populations from different temperature regimes (Svalbard and Kirkenes, Norway). Thermal stress responses at 20°C, 24°C and 28°C were assessed by measuring photosynthetic performance and expression of heat shock protein (HSP) genes (*shsp*, *hsp90* and *hsp70*). We detected population-specific responses between the two populations of *F. distichus*, as the Svalbard population revealed a smaller decrease in photosynthesis performance but a greater activation of molecular defence mechanisms (indicated by a wider repertoire of HSP genes and their stronger upregulation) compared with the Kirkenes population. Although the temperatures used in our study exceed temperatures encountered by *F. distichus* at the study sites, we believe response to these temperatures may serve as a proxy for the species’ potential to respond to climate-related stresses.

## 1. Introduction

Intertidal species experience a broad range of abiotic and biotic environmental factors that vary considerably in magnitude [1]. Particularly during low tides, these species are subjected to stresses that approach their physiological tolerance limits [1–3]. For instance, most intertidal species generally live close to their upper thermal tolerance limits and have low ability to respond to further rising temperatures [3–5]. Therefore, intertidal species can be especially susceptible to environmental stresses as was demonstrated by several studies detecting significant changes in their abundance and range limits in response to climatic (temperature) fluctuations [6–8].

Temperature ultimately determines species’ distributional range from microhabitat to biogeographic scales [9] by influencing organismal performance and functioning at molecular and physiological levels [10]. As environments can act as evolutionary forces and result in local adaptive changes in populations [11], local and global (latitude) temperature gradients and variations can shape thermal tolerance of species populations. Thus, differences in thermal tolerance among northern and southern populations may reflect phenotypic plasticity of species or adaptation to natural selection. In addition, organisms from highly variable environments generally differ in their thermal tolerance (including heat shock response, HSR) from congeners in more moderate and stable thermal environments [12].

HSR is one of the mechanisms of thermal tolerance [5,9] and largely depends on expression of heat shock protein (HSP) genes [5,11]. Most HSPs are molecular chaperones that help organisms to ameliorate stress-induced changes by refolding denatured cellular proteins and degrading/replacing proteins that cannot be repaired [13]. Thus, HSPs are the universal biomarkers of environmental stress, especially for non-model species [14]. Five major families of HSPs are conservatively recognized and named according to their molecular weight in kilodalton: the HSP70 (DnaK) family, the chaperonins (GroEL and HSP60), the HSP90 family, the HSP100 (Clp) family and the small HSP (sHSP) family [15]. Among these families HSP70, HSP90 and sHSP are the best studied [11]. The HSP70 family is considered to be the most conserved in all taxa, while sHSPs are the most prevalent in plants [15]. Although *hsp* genes are expressed as a general response to a variety of physiological stressors [16], the best-studied response is thermal activation, which usually occurs in response to temperature increases of 5°C–10°C greater than the average environmental temperature experienced by an organism [17]. However, the character of this response is not a simple on/off state, but a finely tuned process reflecting level of stress tolerance and experienced environmental stress [11,16].

Gene expression profiling is one of the approaches to assess the ability of an organism to respond to environmental stress [18] and, therefore, to gain an insight into species-specific physiological acclimation, adaptation to environmental conditions and tolerance limits [19]. For example, wide plasticity of *hsp* gene expression patterns has been observed in ectothermic organisms, ranging from short-term acclimation (including seasonal variation) to evolutionary timescales [20]. Intraspecific studies of variation in gene expression have detected locally adapted populations [21–23], maladapted populations from the range edges [24,25] and species that appear to lack HSR as a result of extreme adaptation/specialization to constant cold temperatures [14,26,27].

Thermal tolerance and stress conditions in photosynthetic organisms can also be assessed by photosynthetic performance, as photosynthesis is specifically sensitive to thermal stress [28]. Photosynthetic performance, in turn, can be evaluated by chlorophyll *a* fluorescence measurements [29], specifically the maximum quantum yield (*F*_v_/*F*_m_) and performance index (PI_ABS_). Although *F*_v_/*F*_m_ only represents the functionality of photosystem II [30], it remains a commonly used measurement of stress levels in plants (e.g. [31]) and seaweeds, including *Fucus* spp. (e.g. [25,32]). The performance index PI_ABS_, however, is a multi-parametric expression of three independent functional steps of photosynthesis (density of active reaction centres, excitation energy trapping and conversion of excitation energy to electron transport) [33] and reflects the functionality of both photosystems I and II [30]. Consequently, PI_ABS_ is a rapid and sensitive measure of physiological performance (vitality) of photosynthetic organisms under stress conditions [34] and outperforms *F*_v_/*F*_m_ [35].

Intertidal sessile organisms with a wide distributional range are excellent models for determining the variation in thermal stress response in species with latitudinally separated populations living at different temperature regimes. Brown algae of the genus *Fucus* (Heterokontophyta; Fucaceae) are habitat-forming primary producers and among the most abundant organisms on intertidal rocky shores in the Northern Hemisphere [36]. The genus originated in the north Pacific and after the opening of the Bering Strait colonized the north Atlantic at least twice with radiation into two distinct lineages: Lineage 1 including *Fucus distichus*, *Fucus serratus* and others; and Lineage 2 including *Fucus spiralis*, *Fucus vesiculosus* and others [37,38]. Only the cold-adapted*F. distichus* displays a wide latitudinal and longitudinal distribution along Arctic and subarctic coasts of both the north Atlantic and north Pacific Oceans and its phylogeography and genetic structure are well characterized [36–38]. Although maximum sea surface temperature was identified as the most important factor restricting the fundamental niche of *F. distichus*[39], no study has profiled photosynthetic performance and gene expression in response to thermal stress among different populations of *F. distichus*, in contrast to the congeners *F. serratus* [40] and *F. vesiculosus* [25]. Here, we investigated the thermal stress response in two populations of *F. distichus* by examining photosynthetic performance and expression of selected *hsp* genes. Although the temperatures of the thermal stress experiments in our study (20°C, 24°C, 28°C) exceeded mean temperatures encountered by *F. distichus* throughout its distributional range, they are important for identification of a population’s thermal tolerance; thus serving as a proxy for their responses to climate-related stresses, in particular to predicted extreme and short-term heat wave events [41]. We tested the hypotheses that: (i) thermal stress responses differ between populations exposed to a cold and stable versus warmer and variable temperature regimes, and (ii) individuals from a population exposed to a cold and more stable temperature regime will display lower thermal tolerance and experience higher stress.

## 2. Material and methods

### 2.1 Set-up and experimental design

**Figure 1.**
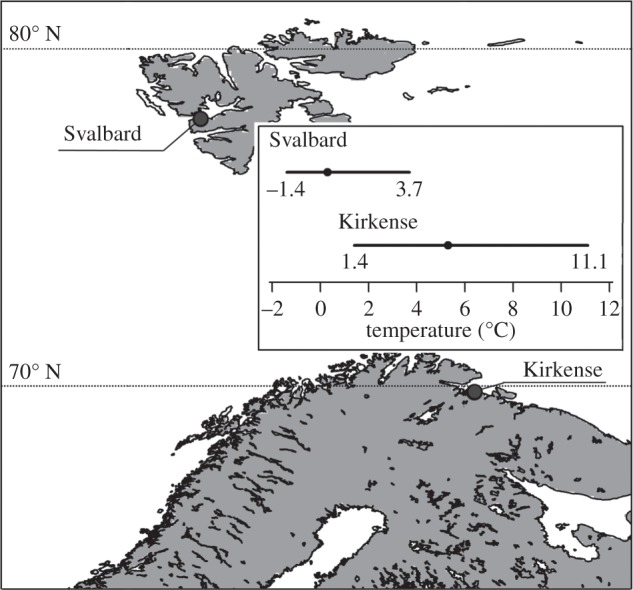
Locations and temperature conditions of sampled *Fucus distichus* populations. Temperature condition is sea surface temperature accessed via the Bio-Oracle dataset (http://www.bio-oracle.ugent.be; [42]), mean is depicted as a dot, range as line.

Populations of *F. distichus* were sampled at two locations in northern Norway, which differed in average sea surface temperature: Barentsburg, Svalbard (hereafter, Svalbard; 14.2457° E, 78.3509° N) and Grense-Jakobselv, Kirkenes (hereafter, Kirkenes: 30.7694° E, 69.7902° N; figure 1). Adult individuals were collected in late May–early June 2011 and transported in cooling boxes with icepacks to the Mørkvedbukta facilities of the University of Nordland (Bodø, Norway) within 24 h of collection. The intact individuals were attached to rubber mats and acclimated in an aquarium (1×1×0.3 m) with aerated and re-circulating natural filtered seawater at 8°C without duplicating a tidal cycle. Acclimation proceeded for 10 weeks in common-garden conditions under low photosynthetic photon flux density (on average 50 μmol m^−2^ *s*^−1^) and a 16 L : 8 D regime [43].

Three thermal stress experiments were conducted in a common-garden format at 20°C, 24°C and 28°C in a thermostatically controlled and aerated water bath (cooled incubator SANYO MIR 154, SANYO Electric Co., Ltd.). Replicated apical tips from each of six to 10 individuals were used for each of the three thermal stress experiments that were performed successively between September and November 2011. Chlorophyll fluorescence measurements and samples for real-time quantitative polymerase chain reaction (qPCR) analysis were collected at acclimation conditions at 8°C (control), after 15 and 60 min of thermal stress, and after 24 h recovery at 8°C (in total three treatments).

### 2.2 Stress response assessment

Photosynthetic response to thermal stress was assessed by chlorophyll fluorescence measurements, specifically the maximum quantum yield (*F*_v_/*F*_m_) and performance index (PI_ABS_). Chlorophyll fluorescence measurements were collected with the fluorometer FluorPen FP100max (Photon Systems Instruments) after a 15 min dark-adaptation period; three replicates for each tip were averaged. The performance index and *F*_v_/*F*_m_ were calculated from O-J-I-P parameters using the FluorPen software.

Samples for real-time qPCR analysis were excised directly after fluorescence measurements from each of the control and treatment samples (0 min, 15 min, 60 min, 24 h) of the 24°C thermal stress. Apical tissue was immediately frozen in liquid nitrogen, subsequently lyophilized and stored at −80°C. Total RNA was extracted according to the protocol described elsewhere [44]. RNAs were cleaned using the RNeasy^®^ MinElute^®^ Cleanup kit (Qiagen^®^) and quantified with Nanodrop^®^ ND 1000 spectrophotometer (Thermo Fisher Scientific Inc.), while RNA integrity was verified by agarose gel electrophoresis. Reverse transcription was performed using QuantiTect^®^ Reverse Transcription kit (Qiagen) with a starting quantity of 350 ng of total RNA from each sample. qPCR primers (amplicon size of 75–105 bp) were designed using the Primer Express v. 3.0 software for three target genes: (i) *sHsp* (small HSP, EU780018.1) (F: 5′-AGCGTGGTTACTCCTTCA-3′, R: 5′-CCGTCTTCATCTCCTGGT-3′), (ii) *hsp70* (EU780017.1) (F: 5′-GGGTGCTTATCCAGGTGTA-3′, R: 5′-CCGTCCAGGTTGAACTTG-3′), (iii) *hsp90* (EU780016.1) (F: 5′-GGTCGCATTCACAGGCTTATC-3′, R: 5′-CGTCCTCTCCGTCGTCTC-3′), and two reference genes: (iv) *actin* (U11697.1) (F: 5′-AGCGTGGTTACTCCTTCA-3′, R: 5′-CCGTCTTCATCTCCTGGT-3′) and (v) *elongation factor* (GH700727.1) (F: 5′-CCGCTACAAGGAGATCAAGGA-3′, R: 5′-AGATGGGCACGAAGGGAAT-3′).

Duplicated qPCR reactions were performed in a total volume of 10 μl, using a LightCycler^®^ 480 (Roche Applied Science) with SYBR Green chemistry (LightCycler 480 SYBR Green I Master, Roche Applied Science). Primers were used in a final concentration of 7.5 μM each. The amplification protocol was: 95°C for 10 min, 50 cycles of 95°C for 10 s, 64°C for 20 s and a fluorescence collection at 72°C. At the end of the qPCR, melting curve analysis was performed in order to verify amplification specificity. The amplification efficiency of PCR was calculated from dilution curves (1 : 5 dilution per step) using a pooled cDNA mix of all populations from all treatment conditions. Efficiencies were calculated from the slope of the threshold cycle (Ct) versus cDNA quantity plot in the standard way from *E*=10^−1/slope^, where *E* stands for the amplification efficiency [45]. A normalization factor of gene expression for each population was calculated with two reference genes using geNorm 3.5 [46].

### 2.3 Data analysis

Data analysis was performed with R v. 2.13.1 [47]. Normal distribution of data on photosynthesis performance and relative gene expression were assessed visually by frequency histograms and Q–Q plots, while homogeneous variance of data was checked with Cochran’s C test. As not all the data satisfied parametric assumptions, non-parametric rank methods were applied that are generally more robust to outliers and small sample sizes [48]. Longitudinal data (repeated measurements over time on each of several individuals) of photosynthetic performance (PI_ABS_ and *F*_v_/*F*_m_) and gene expression were analysed with the R package nparLD [48] designed for non-parametric analysis of longitudinal data in factorial experiments.

Differences in PI_ABS_ and *F*_v_/*F*_m_ control values were evaluated between populations (a whole-plot factor that stratifies samples in independent groups) and different experiments (as time factor, a sub-plot factor that stratifies repeated measurements). Differences in gene expression under control conditions between two populations were assessed with a Mann–Whitney *U*-test for each gene separately. For each thermal stress experiment, differences in PI_ABS_, *F*_v_/*F*_m_ and relative gene expression (only the thermal stress at 24°C) values between populations and treatments (control, 15 min of thermal stress, 60 min of thermal stress and 24 h recovery) were assessed using a two-factorial layout with population and treatment (time factor) as fixed factors. In case of significant results, a one-factorial layout with treatment effect was applied to each population separately, followed by Dunnett’s-like multiple pairwise comparisons (control—15 min thermal stress, control—60 min thermal stress, control—24 h recovery) with subsequent Bonferroni correction of ANOVA-type statistics (ATS).

## 3. Results

### 3.1 Photosynthetic performance

**Figure 2.**
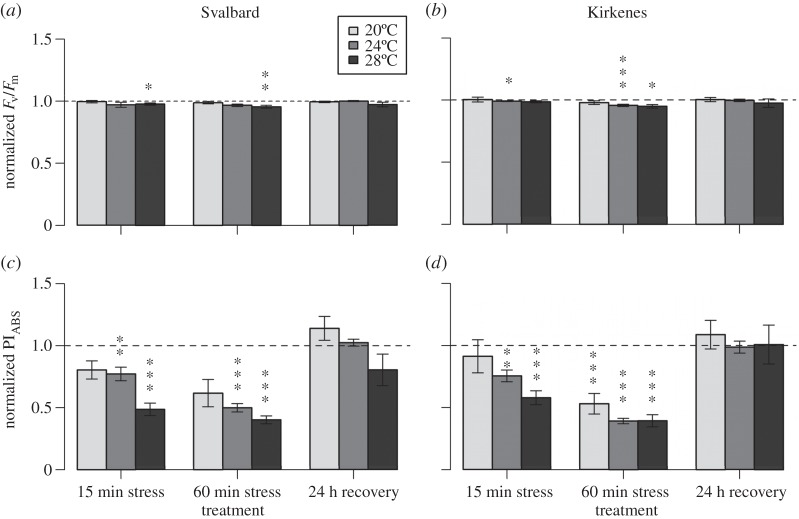
Response of two populations of *Fucus distichus* to short-term thermal stress. The response was measured at three temperatures by maximum quantum yield (*F*_m_/*F*_v_) (*a*,*b*) and the performance index (PI_ABS_) (*c*,*d*) for the Svalbard (*a*,*c*) and Kirkenes (*b*,*d*) populations. All values under treatment conditions (mean±s.e.) were normalized to values under control condition (control level =1). Significant differences between control values and values under treatment conditions are indicated by asterisks over treatment condition (*p<0.05, ^**^p<0.01, ^***^p<0.001).

Values of PI_ABS_ and *F*_v_/*F*_m_ under control conditions did not differ significantly between the two populations and the three experiments (PI_ABS_: population, ATS_(1)_=1.052, *p*=0.305; experiment, ATS_(2.5)_=1.74, *p*=0.167; population : experiment, ATS_(2.5)_=0.60, *p*=0.581 and *F*_v_/*F*_m_: population, ATS_(1)_=0.826, *p*=0.363; experiment, ATS_(2.7)_=1.60, *p*=0.193; population : experiment, ATS_(2.7)_=0.86, *p*=0.454) and equal to 0.84±0.03 (mean±s.e.) and 0.70±0.00, respectively. However, a two-factorial analysis of photosynthetic response of *F. distichus* to thermal stress had a significant effect of treatment (*p*<0.001) at all three temperatures except *F*_v_/*F*_m_ response at 20°C stress (table 1). The only significant effect of population was detected under 24°C of thermal stress in terms of the PI_ABS_ measure and no interaction between population and treatment factors was found (table 1). Nevertheless, photosynthetic response of the Kirkenes population was affected at lower stress temperatures (24°C for *F*_v_/*F*_m_, figure 2*b*; 20°C for PI_ABS_, figure 2*d*) than of the Svalbard population (28°C for *F*_v_/*F*_m_, figure 2*a*; 24°C for PI_ABS_, figure 2*c*). In addition, the PI_ABS_ (figure 2*c*,*d*) measure of photosynthetic performance was more sensitive to stress than the *F*_v_/*F*_m_ measure (figure 2*a*,*b*). Thus, PI_ABS_ was significantly decreased from 20°C, whereas *F*_v_/*F*_m_ was affected only from 24°C. However at 24°C and 28°C stresses, a strong decrease in PI_ABS_ values reveal no difference in response between two populations, unlike *F*_v_/*F*_m_ which did.

**Table 1.** Results of two-factorial non-parametric analysis of *F. distichus* response to thermal stress at three different temperatures. (Results are presented in format ‘ANOVA-type statistics_(*d*.*f*.)_ *p*-value’ (n.s., non-significant). *p<0.05,^**^p< 0.01,^***^p<0.001.)

	effect		
response variable	population	treatment	population : treatment
HS 20°C			
PI_ABS_	1.02_(1)_ (n.s.)	12.67_(2.4)_***	0.39_(2.4)_ (n.s.)
*F*_v_/*F*_m_	0.22_(1)_ (n.s.)	1.10_(2.4)_ (n.s.)	0.20_(2.4)_ (n.s.)
HS 24°C			
PI_ABS_	21.28_(1)_***	51.41_(2.3)_***	0.23_(2.3)_ (n.s.)
*F*_v_/*F*_m_	0.06_(1)_ (n.s.)	9.80_(2.5)_***	0.56_(2.5)_ (n.s.)
*shsp*	41.03_(1)_***	110.36_(2.5)_***	3.11_(2.5)_*
*hsp70*	26.73_(1)_***	22.36_(2.8)_***	1.84_(2.8)_ (n.s.)
*hsp90*	38.52_(1)_***	7.81_(2.3)_***	0.78_(2.3)_ (n.s.)
HS 28°C			
PI_ABS_	0.14_(1)_ (n.s.)	32.84_(2.2)_***	0.45_(2.2)_ (n.s.)
*F*_v_/*F*_m_	0.48_(1)_ (n.s.)	6.58_(2.4)_***	0.39_(2.4)_ (n.s.)

At 20°C thermal stress, PI_ABS_ and *F*_v_/*F*_m_ values did not differ from control values in the Svalbard population during all treatments, whereas the Kirkenes population showed significant reduction in PI_ABS_ after 60 min of stress (*p*<0.001) with recovery after 24 h (figure 2*a*–*d*). Under the stress of 24°C, the pattern of PI_ABS_ response was similar between two populations: significant decrease of the performance index after 15 min of stress (*p*<0.05) with continuing reduction after 60 min of stress (*p*<0.001), and recovery to control values after 24 h. Significant effect of population factor during the 24°C thermal stress was found only at 60 min of stress and expressed by lower PI_ABS_ values in individuals from Kirkenes compared to Svalbard. In terms of *F*_v_/*F*_m_ response, the Svalbard population was not affected by any of 24°C stress treatments, whereas individuals from Kirkenes showed a highly significant reduction of *F*_v_/*F*_m_ after 15 and 60 min of stress (*p*<0.05 and *p*<0.001). Changes in PI_ABS_ at 28°C thermal stress were detected as for 24°C stress: a significant decrease after 15 and 60 min of stress in both populations (*p*<0.0001) with full recovery after 24 h. A similar pattern of changes in *F*_v_/*F*_m_ was revealed for the Svalbard population, while the Kirkenes population was affected only after 60 min of stress.

### 3.2 Gene expression

**Figure 3.**
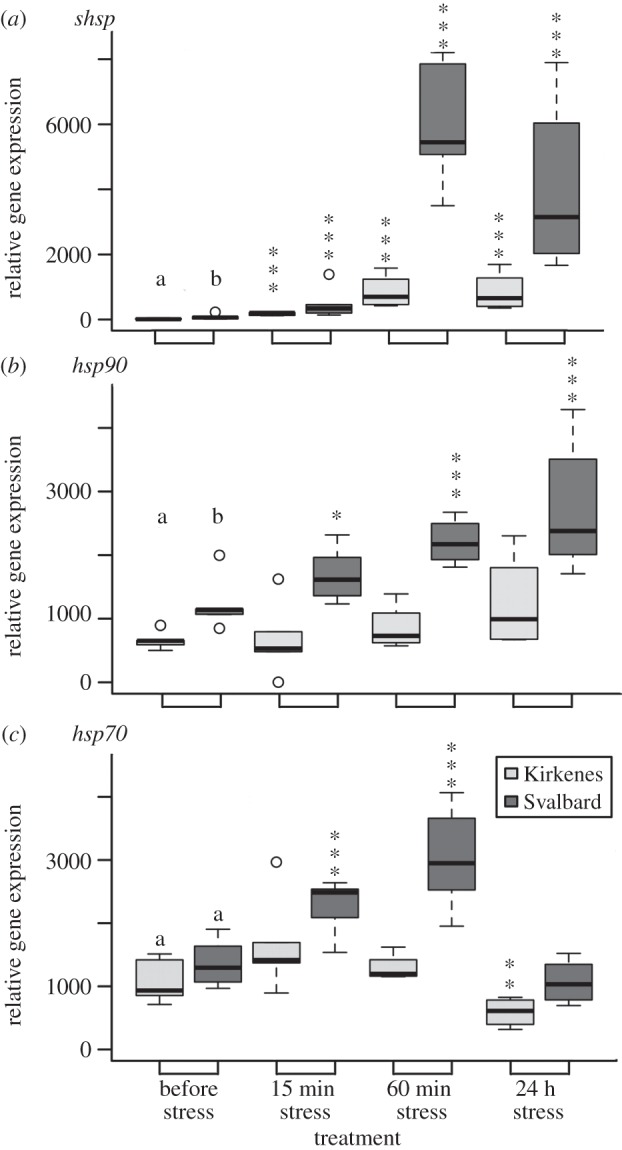
(*a*–*c*) Response of the two populations of *Fucus distichus* to short-term 24°C thermal stress in relative gene expression of hsp genes. Lower case letters (a,b) indicate significantly different expression levels (*p*<0.05) between the populations before stress (i.e. control conditions). Significant differences between control values and values under treatment conditions are indicated by asterisks over treatment condition (*p<0.05,^**^p<0.01,^***^p<0.001). Open circles are outliers.

Expression level of the *hsp70* gene under control conditions during the 24°C thermal stress did not differ significantly between the two populations (Kirkenes, 1100.71±127.78; Svalbard, 1367.81±137.46, *W*=12, *p*=0.128; figure 3*c*). However, the Svalbard population had significantly higher expression levels than the Kirkenes population for both *shsp* (73.97±28.53 versus 18.36±7.44; *W*=8, *p*=0.038) and *hsp90* genes (1204.02±138.11 versus 648.50±46.77; *W*=1, *p*=0.001; figure 3*a*–*b*).

Highly significant effects of population and treatment factors were found for all three *hsp* genes; the only significant interaction between two factors was detected for the *shsp* gene (table 1). Overall, the thermal stress expression levels for all genes were higher in the Svalbard population than in the Kirkenes population (figure 3). Expression of *shsp* under 24°C was significantly (*p*<0.001) upregulated in both populations at 15 and 60 min of stress and after the 24 h recovery phase (figure 3*a*). Gene expression of *shsp* relative to the control for individuals from Svalbard showed a sixfold increase after 15 min of thermal stress, a 82-fold increase after 60 min and a 56-fold higher level than the control at the recovery phase. The Kirkenes population showed a 10-fold increase after 15 min of stress and a stable 46-fold increase of *shsp* expression after 60 min of stress and the 24 h recovery. Levels of *hsp70* gene expression were twofold higher (*p*<0.001) relative to control conditions at both 15 and 60 min thermal stress phases in the Svalbard population, but twofold lower (*p*<0.01) in the Kirkenes population at the 24 h recovery phase (figure 3*c*). Only Svalbard individuals showed a significant increase in the level of *hsp90* transcripts during the experiment treatments, with a maximum twofold upregulation during the recovery phase (figure 3*b*).

## 4. Discussion

A plethora of studies using gene expression profiling has focused on the responses of species and populations along environmental gradients and to various physiological stresses [4,9,49–52]. Based on studies of single species with several populations along a thermal gradient, a general trend emerges: upregulation of *hsp* genes is activated at a lower temperature in organisms from cold habitats than in those from warmer ones [10]. This also implies that organisms from cold habitats will be more stressed (upregulation of *hsps* is more intensive and includes activation of other *hsp* genes) than those from warmer habitats in response to the same stress temperature. The pattern was revealed in killifish *Fundulus heteroclitus*[21,22], copepod *Tigriopus californicus* [23], sea urchins *Strongylocentrotus purpuratus* [53] and eelgrass *Zostera marina* [54].

Although both the Svalbard and Kirkenes populations displayed reduced photosynthetic performance (both *F*_v_/*F*_m_ and PI_ABS_), the reduction was less in the Svalbard population and the molecular response was stronger (indicated by exclusive upregulation of *hsp70* and *hsp90*, and stronger upregulation of *shsp*). The opposing results of the two stress indicators could have two explanations: (i) the indicators are not correlated and assess different cellular components of the stress response as was found in *F. serratus* [40], or (ii) greater upregulation of *hsp* genes may protect and support functioning of photosynthetic apparatus as was shown for some HSPs from the chloroplast stroma in tomato (*Lycopersicon*) [55]. Furthermore, HSPs and photosynthesis may be intimately linked, as upregulation of HSPs is energetically costly [16] and requires energy production from photosynthesis. In addition, cellular components other than *hsps* can be involved in increased thermotolerance of photosynthesis. For example, detoxifying enzymes may protect photosystem II from damage by reactive oxygen species, while osmolyte accumulation (e.g. glycinebetaine) enhances the stability of photochemical performance in photosynthetic apparatus under heat stress [56]. Overall, our initial hypothesis (i) of differential HSR in populations from different temperature regimes holds true for *F. distichus* as well as for the congeners *F. serratus*[40] and *F. vesiculosus* [25,43]. Population-specific differences in the HSR in these *Fucus* species may indicate different thermal tolerances and thermal adaptations to different thermal environments (e.g. mean and range of sea surface temperature); however, a clear resolution of adaptation versus plasticity is hampered by several obstacles (e.g. maternal effect, unknown underlying genetic architecture, etc.).

In the genus *Fucus*, including *F. distichus*, each *hsp* gene reveals specific expression patterns after stress conditions. The gene *shsp* was rapidly upregulated (more than 50-fold), while expression of *hsp70* and *hsp90* was upregulated to approximately twofold depending upon stress conditions and species tolerance ([25,40,43]; figure 3). Small heat shock proteins (sHSPs) are the first protective response to stress (hence the rapid upregulation), but are unable to refold non-native proteins. Instead, they have a high capacity to bind and stabilize non-native proteins, thereby facilitating subsequent refolding by ATP-dependent chaperones, including HSP70 and HSP90 [15]. Under stress HSP70 and HSP90 act as part of a multichaperone machinery to assist the refolding of denatured proteins and elimination of damaged proteins [13]. The significant upregulation of both *hsp70* and *hsp90* genes at 24°C only in the Svalbard population (figure 3) therefore, suggests greater cellular damage compared with the Kirkenes population and proximity to the thermal tolerance limits [19]. Thus, it confirms our hypothesis (ii) that the Svalbard population experiencing colder and nearly half the range of sea surface temperature (5.1°C) than Kirkenes (9.7°C) displayed lower thermal tolerance limits. Although the Svalbard and Kirkenes populations decreased photosynthetic performance after 15 min of stress at 28°C and subsequently recovered (figure 2), the effects of slightly higher than normal temperatures (i.e. 15°C–20°C) for a longer time may be detrimental as shown for the eelgrass *Z. marina* [54,57]. Moreover, a thermally stressful environment increases an organism’s maintenance costs due to production of HSPs and may result in a decrease in overall fitness [11].

Response to changing climate also can be influenced by the genetic diversity of a population [58]. For example, increased genetic diversity in populations of the eelgrass *Z. marina* increased the heat stress resilience and had a positive effect on shoot density and recovery of the entire associated ecosystem [59]. However, for a selfing (hermaphrodite) species such as *F. distichus*, inbreeding and lower genetic diversity could reduce the adaptive potential to respond to climate change. Unfortunately, data on genetic diversity are absent from our Svalbard study site, but are available for the northernmost and southernmost populations of Svalbard, as well as Kirkenes [38]. Allelic richness (six microsatellite loci) in Svalbard populations ranged from 2.13 to 2.30, nearly twofold higher than at Kirkenes (1.41) [38]. Thus, there may be some genetic potential of the Svalbard population to weather the impact of climate change, while for the Kirkenes population it is uncertain if present adaptation or plasticity of the Kirkenes population to the wide range of temperatures will be sufficient to cope with coming climate change.

## 5. Conclusion

Ongoing climate change has already altered natural communities and poses real threats to the persistence of many populations, particularly in the Arctic. An organism’s physiology, genetics, environmental tolerance limits and habitat requirements, are all integrated into a species’ vulnerability to climate change. Results of our thermal stress experiments showed a differential response to elevated temperatures between representatives of Arctic and subarctic populations of *F. distichus* and may indicate locally adapted populations. Such local adaption will probably play an important role during climate-related distributional changes of *F. distichus* and for the whole Arctic intertidal ecosystem.

## Data accessibility

Data file on photosynthetic performance and gene expression from this study can be found in the Dryad database (http://dx.doi.org/10.5061/dryad.t1tk4).
